# Spatial Distribution, Source Apportionment and Risk Assessment of Heavy Metal Pollution in Typical Redevelopment Sites in Pudong New District, Shanghai

**DOI:** 10.3390/toxics14040315

**Published:** 2026-04-08

**Authors:** Cheng Shen, Jian Wu, Ye Li

**Affiliations:** 1 State Environmental Protection Engineering Center for Urban Soil Contamination Control and Remediation, Shanghai Academy of Environmental Sciences, Shanghai 200233, China; shencheng@saes.sh.cn; 2 State Environmental Protection Key Laboratory of Environmental Risk Assessment and Control on Chemical Process, School of Resources and Environmental Engineering, East China University of Science and Technology, Shanghai 200237, China; 3 Shanghai Technology Center for Reduction of Pollution and Carbon Emissions, Shanghai 200235, China; 4 Key Laboratory of Geographic Information Science (Ministry of Education), School of Geographic Sciences, East China Normal University, 500 Dongchuan Road, Minhang District, Shanghai 200241, China

**Keywords:** soil heavy metals, spatial distribution, source apportionment, random forest prediction, health risk assessment

## Abstract

To investigate the characteristics and health risks of heavy metal (HM) contamination in soils of typical industrial sites during urban renewal, this study selected Pudong New District, Shanghai, as a case. Seven HMs (Cd, Pb, Cu, Zn, Ni, Hg, and As) were analyzed for their concentrations, ecological risks, spatial patterns, and potential sources. Inverse Distance Weighted (IDW) interpolation was used to assess spatial distribution, Random Forest (RF) regression to predict HM concentrations, and a two-dimensional Monte Carlo simulation to evaluate human health risks. The results showed that all HMs except As exceeded Shanghai background values in surface soils, with varying levels observed in subsoil and saturated layers. The Index of Geoaccumulation (Igeo) and Risk Index (RI) indicated low contamination and moderate ecological risk. Pearson correlation combined with Positive Matrix Factorization (PMF) identified four major sources: traffic emissions dominated by Cd and Zn, combustion-related sources dominated by Pb and Hg, industry-related inputs dominated by Cu and Ni, and a natural source dominated by As. The RF model demonstrated strong predictive accuracy for Cd, Pb, Hg, and As (R^2^ = 0.80–0.94), and predicted values were consistent with observations. Monte Carlo results showed that non-carcinogenic risks for children and adults were within acceptable limits, while carcinogenic risks reached “notable” levels with probabilities of 62.06%, 55.65%, and 22.49% for children, adult females, and adult males, respectively. Cd and As were identified as key contributors. This work provides scientific support for soil pollution prevention and remediation during urban renewal.

## 1. Introduction

Soil plays a dual role in material cycles, functioning both as a “source” and a “sink”, and acts as a vital medium for the transport and retention of various substances. Pollution of soil caused by inorganic (such as heavy metals, HMs) and organic contaminants has become a global environmental concern [1,2]. Over the past four decades, China has experienced severe soil contamination [3], particularly in the Northeast industrial base, the Pearl River Delta, and the Yangtze River Delta. This contamination is closely associated with industrial activities that release large amounts of toxic and harmful substances, including fly ash, sulfur dioxide, HMs, and volatile organic compounds [4,5,6]. Urban renewal is essential for promoting urban spatial restructuring and transformation of cities in China. By improving and optimizing urban spatial structure and functionality, urban renewal can comprehensively enhance municipal facilities and public buildings [7]. The process often involves closing and relocating heavily polluting enterprises in urban centers, and repurposing these areas for residential and commercial uses, thereby fundamentally altering land use patterns. However, historically intensive land development and human activities have left significant soil environmental problems. These legacy sites often contain substantial residual heavy metal pollution, resulting in elevated levels of environmental contamination [8,9]. Heavy metal pollution is both persistent and highly toxic. Its accumulation in soil not only degrades soil quality but also disrupts ecosystems and poses risks to vulnerable populations [10,11]. Therefore, assessing the current status of heavy metals in urban industrial land and conducting risk assessments are critical for mitigating ecological and health risks, ensuring soil safety, and promoting sustainable urban renewal.

Heavy metal pollution in soils typically originates from both natural processes and anthropogenic activities [12]. Natural sources include inherent heavy metals derived from soil parent materials [13]. Anthropogenic sources mainly result from human activities such as coal combustion and mineral mining, excessive use of fertilizers and pesticides, wastewater irrigation, vehicle exhaust emission, and tire abrasion [14,15]. Multivariate statistical methods, including correlation analysis, principal component analysis (PCA), and cluster analysis, are commonly used for source identification [13]. PCA, which extracts uncorrelated principal components through maximizing variance, can infer pollution sources by explaining the dominant information in the original dataset. While PCA effectively identifies the number and order of factors, it cannot quantify the contribution of individual sources. In contrast, quantitative source apportionment such as chemical mass balance (CMB) [16], absolute principal component-multivariate linear regression (APCS-MLR) [17], UNMIX [18], and positive matrix factorization (PMF) models are employed for this purpose. Among these, the PMF model recommended by the U.S. Environmental Protection Agency (USEPA) has gained widespread use for heavy metal sources apportionment [19]. Soil pollution levels are assessed using indices such as the geo-accumulation index, Nemerow pollution index, and potential ecological risk index [20,21]. Human health risks associated with pollutants are often assessed using the health risk assessment (HRA) model, which calculates hazard indices (HI) and total cancer risk indices (TCR) across multiple exposure pathways. However, uncertainties in pollutant concentrations and model parameters, along with fixed values for parameters such as body weight, intake rates, and exposure duration, may result in overestimated or underestimated health risks. To address these limitations, the Monte Carlo simulation—a probabilistic method based on the law of large numbers—has been widely applied in human health risk assessment studies [22,23].

In recent years, with advancements in data availability and computational power, machine learning (ML)—a prominent branch of artificial intelligence (AI)—has been widely applied across diverse research fields, including environmental science [24,25]. Machine learning entails training models on data to automatically identify internal patterns, thereby enabling both prediction and classification. Common algorithms include support vector machines (SVM), multiple linear regression (MLR), backpropagation (BP) neural networks, decision trees (DT), and random forests (RF). These methods provide powerful nonlinear modeling capabilities and strong generalization performance. Among these, RF stands out as a widely used ensemble learning algorithm in environmental applications owing to its high accuracy and strong robustness to outliers and multicollinearity. For example, Ma et al. [26] applied machine learning algorithms for source apportionment of soil HMs and found that the RF algorithm outperformed DT and SVM, providing more comprehensive and accurate results. Similarly, Liu et al. [27] compared LR, RF, and SVM models for predicting soil cadmium (Cd) content in the eastern oasis of the Tarim Basin. Their results demonstrated that the RF model exhibited superior generalization capability and resistance to overfitting. RF constructs multiple decision trees and integrates their predictions, which enhances model robustness and interpretability. In studies on soil HMs pollution, traditional statistical methods often struggle to capture the complex interactions among pollutants and their spatial heterogeneity. ML methods like RF can extract key variables from large, multidimensional datasets, revealing pollutant distribution patterns and potential sources. This capability provides a solid scientific foundation for pollution risk assessment and management strategies. Therefore, integrating machine learning techniques into soil HM research offers considerable theoretical and practical value.

The central and eastern regions of the Yangtze River have historically experienced intensive industrial development and significant industrial wastewater discharge. The Yangtze River Delta Economic Zone is recognized as one of China’s most important economic development regions [28]. Shanghai, located within this region, is the most densely populated city in the delta. Therefore, the ecological risks posed by soil HMs in Shanghai warrant serious attention. However, most current studies on soil environmental quality focus on farmland soils [29] and urban green spaces [30]. In contrast, studies focusing on source apportionment and risk assessment of soil HMs from typical industrial sites in megacities remain limited.

This study aims to collect soils samples from typical industrial sites in the Pudong New District of Shanghai. It measures the concentrations of seven HMs: cadmium (Cd), lead (Pb), copper (Cu), zinc (Zn), nickel (Ni), mercury (Hg), and arsenic (As). The spatial distribution of these metals is analyzed using the inverse distance weighting (IDW) interpolation method. Soil pollution levels are assessed using the geo-accumulation index and the potential ecological risk index. Source apportionment is conducted through correlation analysis and the PMF model. Finally, the RF model is applied to predict the concentrations of the seven HMs. This comprehensive approach provides an in-depth evaluation of HM contamination in soils of typical industrial sites within Pudong New District, Shanghai. The findings aim to support evidence-based strategies for the prevention and remediation of soil HMs pollution in the context of urban renewal.

## 2. Materials and Methods

### 2.1. Study Area

**Figure 1:**
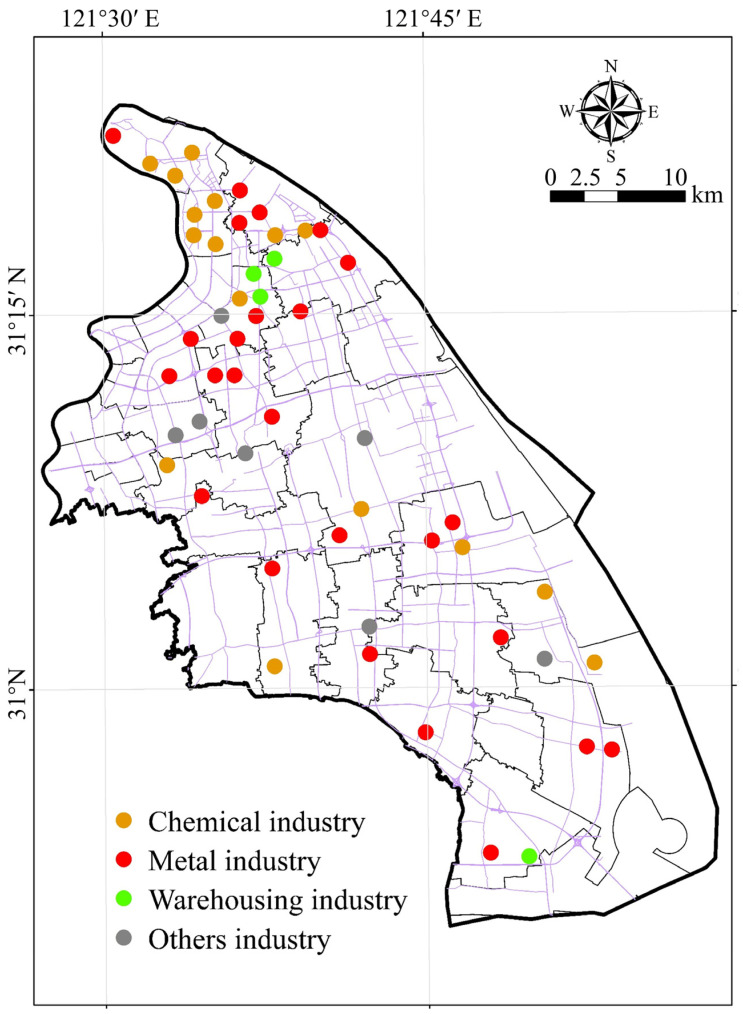
Location distribution of the research sites. The black lines indicate administrative boundaries, and the purple lines indicate the major transportation network.

Pudong New District is located in the east of Shanghai (E 121°27′27″–121°48′43″, N 30°53′20″–31°23′22″). The total area covers 1429.67 km^2^. The parent material for soil formation is primarily Quaternary loose deposits. As Shanghai’s economic engine, Pudong New District has achieved rapid development over the past three decades. In 2023, its regional GDP reached 1.6 trillion yuan, accounting for approximately one-third of Shanghai’s total GDP. However, this rapid economic growth has raised environmental challenges. Industrial clusters contribute to pollutant emissions, dense transportation networks increase carbon emissions, and urbanization compresses ecological spaces, all of which impose significant pressure on the regional soil environment quality. This study examines 52 typical industrial sites: 16 belong to the chemical raw materials and chemical industry (30.8%), 25 to the metal industry (48.1%), 4 to the warehousing industry (7.69%), and 7 sites represent other industry (13.5%). The spatial distribution of these sites is shown in Figure 1.

### 2.2. Sample Collection and Analytical Methods

The study area consists of industrial redevelopment sites in Pudong New District, Shanghai, including chemical manufacturing, metal products, and warehousing sites. Owing to long-term industrial operation and intensive anthropogenic disturbance, including excavation, filling, leveling, and construction, the original soil profiles have been substantially altered. According to the World Reference Base for Soil Resources [31], these soils can be regarded as anthropogenically modified soils, corresponding mainly to Anthrosols and Technosols. In such soils, natural genetic horizons are often absent, mixed, truncated, or difficult to identify; therefore, the soil profile is more commonly characterized by anthropogenic stratification than by clear pedogenic horizonation.

According to the pollution characteristics of 52 typical industrial sites and field investigations, sampling points were established in areas associated with chemical production, the storage and handling of toxic and hazardous substances, wastewater treatment facilities, hazardous waste storage zones, and locations with obvious pollution traces or odors. A total of 252 sampling points were arranged across the redevelopment sites in the study area. In accordance with the Chinese technical guidelines for soil pollution investigation of construction land (HJ 25.1-2019 and HJ 25.2-2019) [32,33], soil samples were collected at three fixed depth intervals: surface soil (0–0.5 m), subsurface soil (0.5–1.5 m), and saturated zone soil (≥1.5 m). This depth-based sampling strategy was adopted because it is the standard approach for contaminated-site investigation in anthropogenically disturbed construction land and is appropriate for evaluating both surface contamination and the vertical migration of pollutants. Heavy metal concentrations in the samples were analyzed following the technical specifications outlined in the “National Soil Pollution Status Detailed Survey—Soil Sample Analytical Testing Methods”, and the soil samples were digested using a microwave-assisted method with a mixture of HCl-HNO_3_-HF (*V*/*V*/*V* = 1:1:1) before analysis [34]. Content of Cd, Pb, Cu, Zn and Ni was measured using an X Series2 inductively coupled plasma mass spectrometer (ICP-MS, Agilent 7700x, Agilent Technologies, Santa Clara, CA, USA). As and Hg concentrations were determined by AFS-3100 dual-channel atomic fluorescence spectrometry (AFS, AFS-9330, Beijing Titan Instruments Co., Ltd., Beijing, China). All experimental water used was ultrapure (China National Pharmaceutical Group Chemical Reagent Co., Ltd., Shanghai, China), consistently of superior grade purity. Quality assurance and quality control (QA/QC) protocols were maintained with soil standard reference materials (GSS-15) [35], blank samples, and duplicates to ensure accuracy and precision. All heavy metal detection limits are shown as in Table S1, and results showed recovery rates for the seven heavy metals ranged from 85.0% to 110.0%, with standard deviations among parallel samples kept below 20%.

### 2.3. Heavy Metal Pollution Assessment

#### 2.3.1. Geo-Accumulation Index Method

The geo-accumulation index (I_geo_) [36], proposed by Helmut Müller in 1969, quantifies heavy metal pollution by accounting for both natural geological background values and anthropogenic influences. This index is categorized into six pollution levels [37,38] (Table 1). The calculation is as follows:

(1) $$I_{geo}={log}_{2}(\frac{C_{i}}{{1.5S}_{i}})$$

In the formula, I_geo_ is the geo-accumulation index for the heavy metal, Ci is the measured concentration of heavy metal i in soil (mg·kg^−1^), and Si is the background value of heavy metal i in soil (mg·kg^−1^). The geo-accumulation index was calculated using the soil background values of Shanghai [36]. The classification standards of geo-accumulation index levels are shown in Table S2.

#### 2.3.2. Potential Ecological Risk Index Method

The potential ecological risk index, introduced by Lars Hakanson in 1980 [39], is a quantitative method used to assess the ecological risk of individual pollutants through a tiered index system. It evaluates both the individual and combined ecological risks of multiple heavy metals. The corresponding calculation formulas are as follows:

(2) $$E_{i}=T_{i}\times (\frac{C_{i}}{S_{i}})$$

(3) $$RI=\sum_{i=1}^{n}E_{i}$$

In the formulas, E_i_ is the potential ecological risk index for heavy metal i, C_i_ is the measured concentration of heavy metal i in soil (mg·kg^−1^), Si is the evaluation standard for heavy metal i (mg·kg^−1^), and RI is the comprehensive potential ecological risk index. Considering the diverse nature of pollutants, the method allows for real-time adjustments [36]. This study uses the soil background values of Shanghai as the evaluation standard (Table S2).

The sum of the toxicity response coefficients for the seven heavy metals (Cd, Pb, Cu, Zn, Ni, Hg, and As) is 96. Referring to previous studies [40,41], the initial threshold value for RI was set at 110 in this study, with each subsequent threshold doubling. The classification criteria for potential ecological risk levels are shown in Table S3.

### 2.4. Positive Matrix Factorization Model

The Positive Matrix Factorization (PMF) model is a source apportionment model recommended by the U.S. Environmental Protection Agency. It decomposes the sample data matrix (X) into the source contribution matrix (g) and source profile matrix (f), along with a residual matrix (e), using a weighted least-squares approach. By utilizing measured pollutant concentrations, the model identifies source composition profiles and quantifies source contributions to observed pollutants at receptor sites [42,43,44]. The calculation formula is represented by the following equation:

(4) $$X_{ij}=\sum_{k=1}^{p}g_{ik}f_{kj}+e_{ij}$$

In the formula, $X_{ij}$ is the concentration of the j-th species in the i-th sample within the sample concentration matrix X, p is the number of pollution sources, $g_{ik}$ is the contribution of the k-th pollution source to the i-th sample, $f_{kj}$ is the concentration of the j-th species in the k-th pollution source, and $e_{ij}$ is the residual matrix.

The model imposes non-negative constraints on matrices G and F (gik ≥ 0 and fkj ≤ 0). The factorization is considered optimal when the weighted sum of squared residuals relative to their uncertainties, denoted as Q, reaches its minimum value. The PMF algorithm determines matrices G and F by continuously minimizing Q. The Q value is calculated as follows:

(5) $$Q={\sum_{i=1}^{n}\sum_{j=1}^{m}\left(\frac{x_{ij}-\sum_{k=1}^{p}g_{ik}f_{kj}}{u_{ij}}\right)}^{2}$$

In the formula, n is the total number of samples, m is the number of different heavy metal elements analyzed, and $u_{ij}$ is the uncertainty of heavy metal element j in the i-th sample. The calculation formula for $u_{ij}$ is as follows:

(6) $$\left(\begin{array}{l} X_{ij}\leq MDL,u_{ij}=\frac{5}{6}*MDL\\ X_{ij}\geq MDL,u_{ij}=\sum \sqrt{{\left(X_{ij}*RSD\right)}^{2}+{\left(0.5*MDL\right)}^{2}} \end{array}\right.$$

In the formula, $X_{ij}$ is the concentration of the j-th heavy metal element in the i-th sample, RSD is the relative standard deviation of heavy metal concentrations, which was set to 0.2 for this study, and MDL represents the method detection limit. The MDL values for Cd, Pb, Cu, Zn, Ni, Hg, and As are 0.03, 0.10, 1.00, 0.50, 5.00, 0.01, and 0.002 mg·kg^−1^, respectively.

### 2.5. Random Forest Model

The RF model, proposed by Breiman, is an ensemble learning algorithm. It belongs to the class of advanced algorithms based on decision trees. It employs bootstrap sampling to randomly draw samples with replacement from the original dataset, generating multiple training subsets. Each subset is used to train an individual decision tree. The outputs from all decision trees are then aggregated to improve the model predictive performance.

In this study, 70% of the 512 samples were randomly assigned as the training set, with the remaining 30% used as the validation set. This approach was used to evaluate the model generalization ability. Model predictive accuracy was assessed using the Root Mean Square Error (RMSE), Mean Absolute Error (MAE), and coefficient of determination (R^2^). The calculation formulas are as follows [26]:

(7) $$MAE=\frac{1}{n}\sum_{i=1}^{n}\left|{C_{i}}^{\prime }-\left.C_{i}\right|\right.$$

(8) $$RMSE=\sqrt{\frac{1}{n}\sum_{i=1}^{n}{\left({C_{i}}^{\prime }-C_{i}\right)}^{2}}$$

(9) $$R^{2}=\frac{\sum_{i=1}^{n}{\left({C_{i}}^{\prime }-{C_{i}}^{\prime \prime }\right)}^{2}}{\sum_{i=1}^{n}{\left(C_{i}-{C_{i}}^{\prime \prime }\right)}^{2}}$$

In the formulas, $C_{i}$, ${C_{i}}^{\prime }$ and ${C_{i}}^{\prime \prime }$ are the measured value, predicted value, and average measured value of heavy metal i in the soil, respectively. Lower RMSE and MAE values, together with an R^2^ value closer to 1, indicate better performance. This evaluation approach effectively improves the accuracy and reliability of modeling soil heavy metal pollution levels.

### 2.6. Human Health Risk Assessment

This study employs a Health Risk Assessment (HRA) model to quantify both carcinogenic risk (TCR) and non-carcinogenic risk (NCR) to humans [45]. The assessment considers three exposure pathways: oral ingestion, dermal contact, and inhalation of soil particles. Recognizing differences in behavior and physiological characteristics among populations, the risk calculations are conducted separately for children, adult males, and adult females. The non-carcinogenic and carcinogenic health risks posed by heavy metals in the soil are evaluated using the Hazard Index (HI) and Total Carcinogenic Risk (TCR). The formulas of HI and TCR are as follows:

(10) $$HI=\sum HQ_{i}=\sum \frac{{ADD}_{i}}{{RfD}_{i}}$$

(11) $$TCR=\sum CR=\sum ADD_{ij}\times SF_{ij}$$

In the formulas, ADD is the average daily dose of heavy metals absorbed by individuals through three exposure routes (ingestion, dermal contact and inhalation). RfDi and $SF_{ij}$ are the reference dose and carcinogenic slope factor for each heavy metal, respectively. The specific values are listed in Table S4 [46,47,48]. HI > 1 indicates a non-carcinogenic risk to human health, whereas an HI ≤ 1 suggests no significant non-carcinogenic effects. For TCR, a value exceeding 1 × 10^−4^ is regarded as unacceptable, whereas a value below 1 × 10^−6^ indicates no significant health risk. When TCR falls between these two thresholds, the carcinogenic risk of human health is considered negligible.

To avoid underestimating or overestimating health risks caused by fixed parameter values in the HRA model, this study integrates the Monte Carlo Uncertainty Analysis Model (MCS) to characterize the probability distribution of health risks from heavy metals in the soil. Detailed exposure parameters utilized in the analysis are listed in Table S5 and the formulas for calculating the average daily intake dose (ADD) are listed in Text S1.

To identify the degree of influence of input parameters on health risk assessment results, this study conducted sensitivity analysis based on Monte Carlo simulation. Use the Spearman rank correlation coefficient in the Oracle Crystal Ball plugin to calculate the correlation between each input variable (including heavy metal concentration and exposure parameters) and the output risk values (HI and TCR). The larger the absolute value of Spearman correlation coefficient, the more significant the contribution of the variable to the risk outcome, and the positive or negative sign indicates the direction of influence. Sensitivity analysis helps to screen key risk factors and dominant exposure parameters, providing priority order for risk management.

### 2.7. Data Analysis

Data processing was conducted using Microsoft Excel 2023 (Microsoft Corporation, Redmond, WA, USA), with measurements below the analytical detection limit recorded as “ND.” Descriptive statistical analyses were performed with SPSS 26.0 (IBM Corporation, Armonk, NY, USA). Spatial distribution maps of soil heavy metals were generated in ArcGIS 10.8 (Environmental Systems Research Institute, Redlands, CA, USA) using the inverse distance weighting (IDW) method. Source apportionment and contribution rates of heavy metals were analyzed using the Positive Matrix Factorization (PMF 5.0) model (U.S. Environmental Protection Agency, Washington, DC, USA). Random forest analysis was carried out in Python 3.9 (Python Software Foundation, Wilmington, DE, USA). Monte Carlo simulations were implemented in Microsoft Excel with the Oracle Crystal Ball add-in (Oracle Corporation, Redwood Shores, CA, USA). Data visualization was performed using Origin 2024 (OriginLab Corporation, Northampton, MA, USA).

## 3. Results and Discussion

### 3.1. Descriptive Statistical Analysis of Soil Heavy Metal Content

The characteristics of seven HMs are summarized in Table 2. The elements Cd, Pb, Cu, Zn, Ni, Hg, and As were detected across various soil depths. Their concentrations generally decreased with increasing soil depth. In surface soil samples, the mean concentrations of Cd, Pb, Cu, Zn, Ni, Hg, and As were 0.17, 30.8, 43.4, 156, 44.7, 0.10, and 8.87 mg·kg^−1^, respectively. The mean levels of Cd, Pb, Cu, Zn, Ni, and Hg exceeded Shanghai background values by factors of 1.33, 1.21, 1.52, 1.81, 1.40, and 1.04, respectively. In subsurface soil samples, the corresponding mean concentrations were 0.15, 27.7, 33.6, 135.5, 35.7, 0.08, and 8.76 mg·kg^−1^. Cd, Pb, Cu, Zn, and Ni were 1.12, 1.09, 1.17, 1.57, and 1.12 times the Shanghai soil background values, respectively. In saturated zone soil samples, only Zn and Ni exceeded these background values.

Compared with other regions worldwide, the levels of heavy metals in soils of the Pudong New Area are generally low. In the industrialized area of Dunkirk, France, surface soil Cd, Zn, and Ni contents ranged from 0.44 to 0.53 mg·kg^−1^, 116 to 132 mg·kg^−1^, and 32.9 to 38.8 mg·kg^−1^, respectively, largely due to the deposition of metallurgical dust. Except for Ni, which was comparable to levels found in Pudong, Cd and Zn concentrations were markedly higher than those in the study area [49]. In Celje, Slovenia, soils surrounding a zinc smelter exhibited severe contamination, with Cd reaching as high as 289 mg·kg^−1^, and Pb and Zn reaching 865 mg·kg^−1^ and 230 mg·kg^−1^, respectively—representing a typical scenario of extreme point-source pollution [50]. In contrast, soil heavy metal concentrations in the copper smelting and steel industrial area of Port Kembla, Australia (Pb: 20 mg·kg^−1^, Zn: 42 mg·kg^−1^, Cu: 49 mg·kg^−1^, and As: 3.20 mg·kg^−1^) were relatively low and comparable to those observed in Pudong [50]. On a global scale, soil heavy metal contamination induced by industrial activities exhibits pronounced regional heterogeneity. While many European industrial zones are characterized by high-intensity point-source pollution, Pudong, as a comprehensive industrial agglomeration area, displays a pattern of multi-source, low-level accumulation—a feature consistent with the general trend observed in newly industrializing regions of Asia.

The coefficient of variation (CV) for HMs in surface soil decreased in the following order: Ni (119%) > Cu (107%) > Cd (100%) > Zn (81.2%) > Pb (69.4%) > Hg (66.3%) > As (19.7%). Except for As, other HMs in surface soil exhibited high variability, indicating the impact of human activities on the region [51]. In subsurface soil, the CVs for Cd, Pb, Cu, Zn, Ni, Hg, and As were 75.1%, 74.7%, 40.7%, 112.9%, 148.8%, 52.1%, and 20.8%, respectively. In saturated zone soils, Cd, Zn, and Ni exhibited significant variability. Overall, the descriptive statistics indicate enrichment of these six HMs (Cd, Pb, Cu, Zn, Ni, and Hg) in the study area’s soils.

### 3.2. Spatial Distribution Characteristics of Soil Heavy Metals

**Figure 2:**
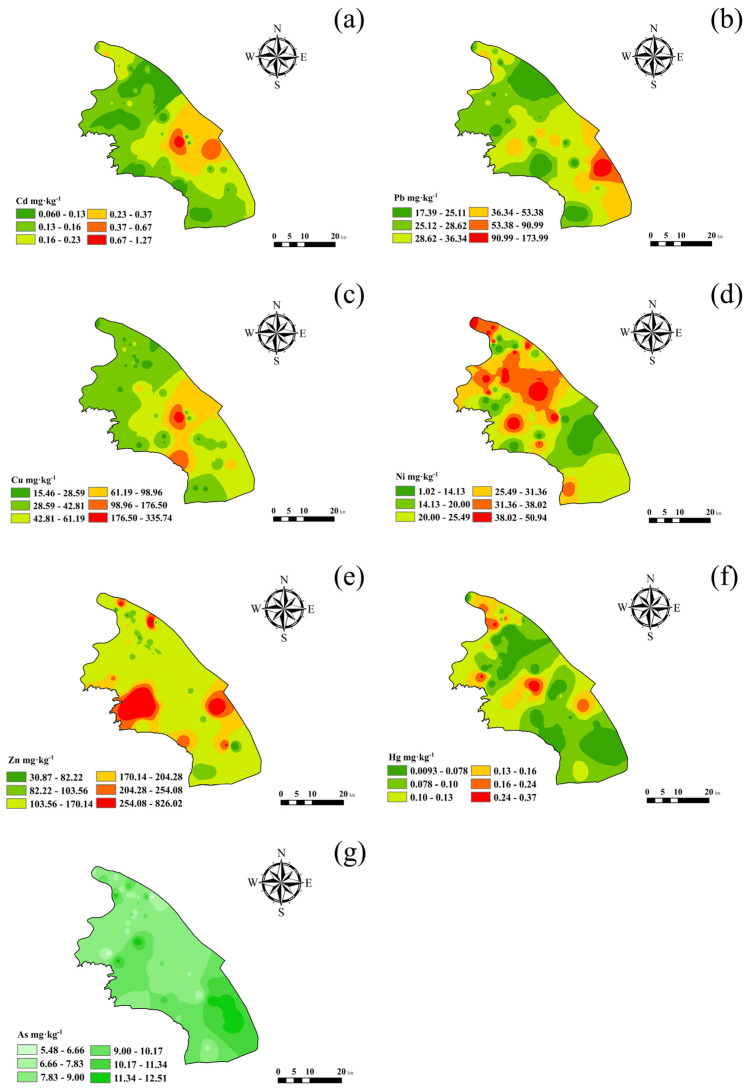
Spatial patterns of heavy metal distribution in the study region: (**a**) Cd; (**b**) Pb; (**c**) Cu; (**d**) Ni; (**e**) Zn; (**f**) Hg; and (**g**) As.

The IDW method was applied to analyze the spatial distribution of HMs in surface soil (Figure 2). The cross validation results (Table 3) indicate that there are differences in the interpolation accuracy of each element: As has the smallest prediction error (RMSE = 1.58 mg·kg^−1^, MAPE = 12.6%), followed by Hg (RMSE = 0.06 mg·kg^−1^, MAPE = 16.4%) and Cd (RMSE = 0.12 mg·kg^−1^, MAPE = 18.3%); The prediction errors of Cu, Zn, and Ni are relatively large (MAPE > 20%), consistent with their high spatial variability (coefficient of variation > 100%). The RMSSE values of all elements are close to 1.0, indicating that the prediction uncertainty estimation of the IDW model is reasonable. The results of spatial interpolation indicate that high concentrations of Pb and Hg are primarily concentrated in the central and eastern part of the study area, exhibiting widespread enrichment. Additionally, scattered patches of elevated Pb occur in the central and southern regions, while high Hg values appear sporadically in the central and northern parts. Cd and Zn are widely distributed throughout the area, generally elevated exhibiting concentrations. Cd shows a broad high-enrichment zone in the eastern region, whereas Zn displays extensive high-value areas in both the eastern and western parts. High concentrations of Cu and Ni are mainly located in the central area, with scattered patches also present in the southern region. As is relatively fairly evenly distributed, with overall lower concentrations. Its high-value areas are mainly in the southern part of the study area, with concentrations decreasing from north to south. The results indicate that Pb and Hg share similar distribution patterns (Figure 2b,f). Cd and Zn are widely distributed (Figure 2a,e), while Cu and Ni exhibit comparable spatial patterns in the central study area (Figure 2c,d).

### 3.3. Environmental Risk Analysis of Soil Heavy Metals

#### 3.3.1. Geo-Accumulation Index Assessment

**Figure 3:**
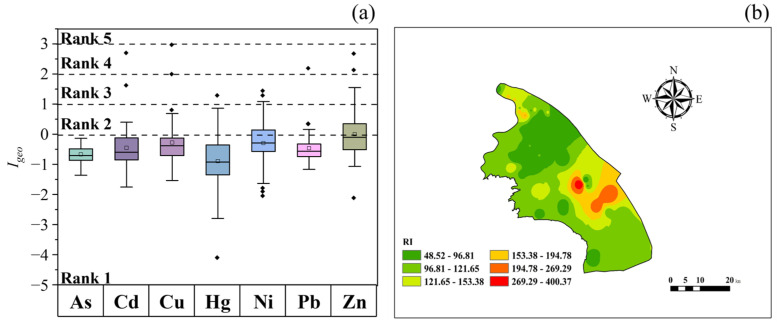
Cumulative Index method for ecological risk assessment: (**a**) I_geo_; (**b**) potential ecological risk space section. The squares represent the mean values, and the diamonds represent outliers.

The results of I_geo_ shown in Figure 3a reveal varying degrees of accumulation among the seven HMs. The mean I_geo_ values ranked as follows: Zn (0.02) > Cu (−0.26) > Ni (−0.27) > Cd (−0.43) > Pb (−0.44) > As (−0.65) > Hg (−0.89). This ranking indicates that Zn exhibits the highest accumulation level, with its mean I_geo_ value approaching the threshold for low pollution (I_geo_ > 0). In contrast, As and Hg show the lowest mean I_geo_ values. For Cd, Pb, Cu, Zn, Ni, and Hg, the proportion of samples classified as no pollution (I_geo_ < 0) accounted for 82.7%, 88.5%, 78.9%, 53.9%, 63.5%, and 82.7%, respectively. Notably, for As, all samples had I_geo_ values below 0, indicating no contamination risk from this element in the surface soils of the study area. For the other six metals, although the mean I_geo_ values were negative, a subset of samples fell into the low to moderately polluted categories, suggesting localized anthropogenic enrichment of these elements. Samples indicating low pollution (0 ≤ I_geo_ < 1) represented 13.5%, 9.62%, 17.3%, 36.5%, 30.8%, and 16.0%, respectively. Moderate pollution (1 ≤ I_geo_ < 2) was observed in 1.92%, 1.92%, 5.77%, 5.77%, and 1.92% of samples for Cd, Cu, Zn, Ni, and Hg, respectively. Samples with moderately severe pollution (2 ≤ I_geo_ < 3) accounted for 1.92%, 1.92%, 1.92%, and 3.85% for Cd, Pb, Cu, and Zn, respectively. Overall, the surface soils show low contamination by Cd, Pb, Cu, Zn, Ni, and Hg, with some samples indicating moderate to moderately severe pollution. This suggests significant accumulation of these heavy metals in the surface soils of the study area.

#### 3.3.2. Potential Ecological Risk Index Assessment

The assessment results of the Potential Ecological Risk Index (PERI) for soils in the study area are presented in Table 3. The mean comprehensive ecological RI is 112, indicating a moderate level of ecological risk. Spatial interpolation of RI, shown in Figure 3b, reveals that areas of high risk concentrate in the eastern part of the study area, with spotty high-risk zones in the central region that warrant attention. The mean potential ecological hazard indices (E_i_) for the metals are ranked as follows: *E_Hg_* (40.7) > *E_Cd_* (40.0) > *E_As_* (9.75) > *E_Cu_* (7.58) > *E_Ni_* (7.00) > *E_Pb_* (6.04) > *E_Zn_* (1.81). Cd and Hg reach severe risk levels, while Cu poses a moderate risk. The remaining metals have E_i_ values below 40, indicating low levels of ecological risk. Cd and Hg pose relatively high potential ecological risks in the study area. In contrast, As presents a low ecological risk, primarily due to its comparatively low concentrations. Other HMs have lower toxicity coefficients, which contribute to their lower potential ecological risks as individual metals. These findings align with previous studies [52,53,54,55]. However, the combined ecological risks posed by multiple HMs warrant further attention. For comparison, soil samples from Jinshan District in Shanghai show an RI of 153, indicating a moderate ecological risk [56], while Minhang District’s RI ranges from 1.86 to 62.3, reflecting a slight ecological risk [57]. The soil RI value in Gangcheng District of Laiwu City is 282.79, in Dagang Industrial Zone of Tianjin City it is 221.12, in the surrounding area of Sichuan Shimian Industrial Zone it is 114.73~452.55, and in Penglai Xianjie Park of Guiyang City it is 124.33~222.00 [58,59,60,61]. This comparison underscores the ecological concerns in Pudong New District, a national-level megacity development zone.

### 3.4. Soil Heavy Metal Source Analysis

#### 3.4.1. Correlation Analysis

**Figure 4:**
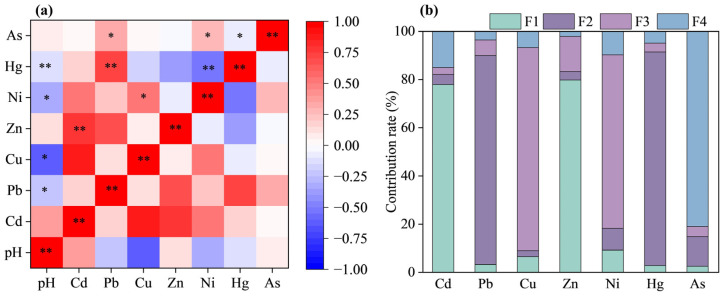
Source analysis of heavy metals in soil: (**a**) Pearson correlation analysis; (**b**) calculation results of the contribution rate of the PMF source resolution model. (Note: ** and * represent *p* < 0.01 and 0.05 levels, respectively).

The correlation analysis results of soil heavy metals are shown in Figure 4a. Soil pH exhibits a significant positive correlation with Pb, Cu, Ni, and Hg (*p* < 0.05), suggesting that Pb, Cu, Ni and Hg may originate from a common source [62]. Cd and Zn display a significant positive correlation (*p* < 0.01), with no significant correlations observed between Cd and other HMs. Pb is significantly correlated with Hg and As (*p* < 0.01); notably, Pb and Hg have a significant positive correlation. Additionally, Cu and Ni exhibit a significant positive correlation (*p* < 0.01).

Correlation analysis is effective in revealing relationships among HMs and can aid in identifying their potential sources. HMs showing correlations often exhibit similar spatial clustering patterns. As illustrated in Figure 2, Cd and Zn are widely distributed, forming high-concentration zones observed along major urban roads except for industrial sites. High concentrations of Pb and Hg are concentrated in the central and eastern part of the study area, while Cu and Ni display similar spatial distributions in the central area. The consistency between spatial clustering and correlation factor analysis supports the inference that Pb, Cu, Ni, and Hg may originate from the same source. In contrast, As shows low correlation with other metals, implying an independent source. The combined results of spatial distribution and correlation analyses provide valuable insights into the potential sources of soil HMs accumulation, forming a foundation for subsequent source apportionment modeling.

#### 3.4.2. PMF Source Apportionment Model

Using the positive definite matrix factorization (PMF) model to analyze the sources of seven heavy metals, multiple iterations were performed with factor numbers ranging from 3 to 6. The results showed that when the number of factors was set to 4, the goodness of fit r^2^ of each element was greater than 0.85, and the residual distribution was concentrated between −3 and +3. The Q (robust)/Q (true) ratio was close to 1.0, indicating that the model had the best fitting effect. The bootstrap analysis (100 iterations) showed that the four factors were successfully identified in over 80% of the iterations, and the 5–95% confidence interval for factor contributions was relatively narrow (figure omitted), indicating good stability of the solution. Sensitivity analysis shows that the source contribution rate is not sensitive to changes in uncertainty weights, and the main source identification results remain stable. The contribution rates of the four source factors analyzed by PMF to each element are shown in Figure 4b.

The source apportionment results revealed that Factor 1 (F1) was predominantly associated with Cd and Zn, which accounted for 77.9% and 79.8%, respectively. Pudong New District, a major transport hub in Shanghai, is an emerging cluster in integrated circuits, biomedicine, software, and IT services. With ongoing economic development, industries such as metal smelting and chemical pharmaceutical manufacturing consume large amounts of energy. Additionally, high traffic volume also contributes to elevated HM concentrations in the study area. Wang et al. [63] previously identified Cd and Zn hotspots in Beijing’s central urban soils, primarily associated with metal product manufacturing and traffic-related pollution. Cd and Zn are widely used in automotive manufacturing for electroplating protective layers that resist corrosion [64]. In a study of farmland soils in the Xijiang River Basin, Song et al. [65] identified road dust from transportation as a major source of Cd. Zn is also commonly used as a tire hardening agent and is present in zinc-plated automotive parts [51,66]. Based on the combined evidence from spatial interpolation, correlation analysis, and principal component analysis, F1 is inferred to represent a “transportation-related source”.

Factor 2 (F2) was mainly characterized by Pb and Hg, contributing 86.7% and 88.6%, respectively. The pronounced variability of Pb and Hg in soils indicates significant anthropogenic influence. Pb and Hg are typical tracer elements for the combustion of fossil fuels, especially coal. Coal naturally accumulates Pb and Hg, which are released in gaseous or particulate form during combustion and accumulate in soil through atmospheric deposition. Industrial dust and exhaust emissions contribute to heavy metal accumulation in soils via atmospheric deposition and leaching [67]. Pb and Hg are primarily associated with the production of organic chemical raw materials and specialized chemical manufacturing. In the central part of Pudong New Area, there are concentrated industrial functional zones such as Waigaoqiao Bonded Zone and Waigaoqiao Port Comprehensive Bonded Zone, forming an industrial cluster with international trade, modern logistics, and advanced manufacturing as its core. Among them, industries such as petroleum product manufacturing, lubricant processing, and chemical product production consume huge amounts of energy, and coal and heavy oil combustion are important features of industrial activities in the region [68,69]. Zhao et al. [70] identified industrial pollution as the main source of Hg and Pb in soils around typical industrial parks upstream of the Yellow River. Chen et al. [71] attributed Pb contamination in residential soils in Shaanxi mainly to fossil fuel combustion. Therefore, F2 is interpreted as a “chemical combustion source.”

Factor 3 (F3) showed the highest contributions from Cu and Ni, at 84.3% and 72.00%, respectively. Cu and Ni exhibited similar spatial distributions, with high-concentration zones concentrated in the central area (Figure 2c,e). This area is characterized by industries including metal surface treatment, heat processing, integrated circuit fabrication, and metal structure manufacturing. Electroplating rinsing processes produce acidic wastewater containing high concentrations of Cu^2+^ and Ni^2+^ ions [72,73]. Electroplating emissions contain metal dust, chromic acid mist, and organic pollutants; acid mist volatilization (polishing) and spray plating are main sources [74]. These airborne pollutants settle onto the surface environment through wet and dry atmospheric deposition, and then infiltrate into deeper soils via rainwater leaching, resulting in deeper HM accumulation. Previous studies by Li et al. [75] confirmed electroplating as the primary source of Cu and Ni emissions over ten years. Jiang et al. [76] also showed metal processing industries produce “three wastes” (wastewater, exhaust gas, and solid waste) containing Cu and Ni that accumulate in soil via atmospheric deposition, leaching, and runoff. Improper disposal of acidic etching solutions, passivation gases, and electroplating sludge exacerbates the spatial heterogeneity of heavy metal distribution in soils [76]. Therefore, F3 is inferred as the “metal-industry-related source.”

Factor 4 (F4) was predominantly associated with As, which accounted for 81.0% of its total contribution. As exhibited no significant contamination or spatial variability, suggesting a natural source related to soil’s parent material [57]. Wu et al. [56] found As in Jinshan industrial area soils mainly originated from the soil’s parent material. Therefore, F4 is identified as the “natural source”.

### 3.5. Soil Heavy Metal Content Prediction Based on Random Forest

**Figure 5:**
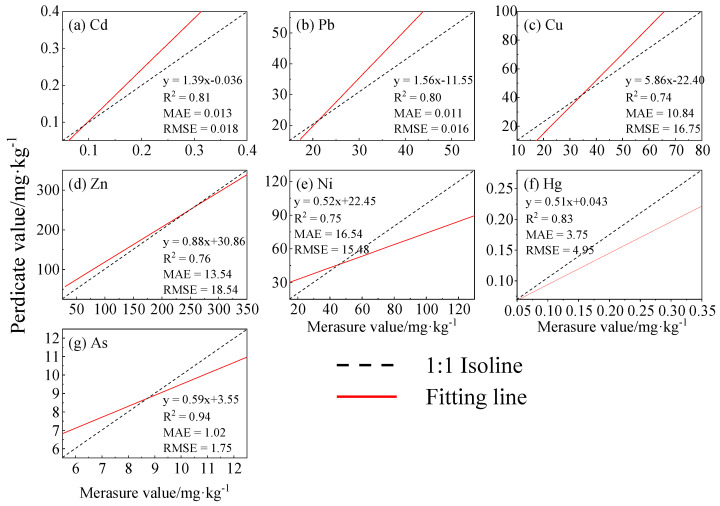
Accuracy results of metal prediction models under the input of the RF model.

The dataset was randomly split into training and validation sets in a 7:3 ratio. The RF model was constructed and trained using the optimal variable set from the training dataset (*n* = 361) along with measured soil concentrations. The optimal variable set from the validation dataset (*n* = 155) was used as input to predict the soil concentrations of these six HMs. Model performance was evaluated using R^2^, RMSE, and MAE metrics. The results are presented in Figure 5a–g. The R^2^ values for the six heavy metals ranked as follows: As (0.94) > Hg (0.83) > Cd (0.81) > Pb (0.80) > Zn (0.76) = Ni (0.76) > Cu (0.74). The RMSE values for As, Hg, Cd, and Pb were 1.75, 4.95, 0.018, and 0.016, respectively, and the MAE values for As, Hg, Cd, and Pb were1.02, 3.75, 0.01, and 0.01. These results indicate that the RF algorithm performs optimally for predicting Hg, Cd, and Pb concentrations. The RF model is robust against noise, has strong generalization ability, effectively captures complex nonlinear relationships, and prevents overfitting.

**Figure 6:**
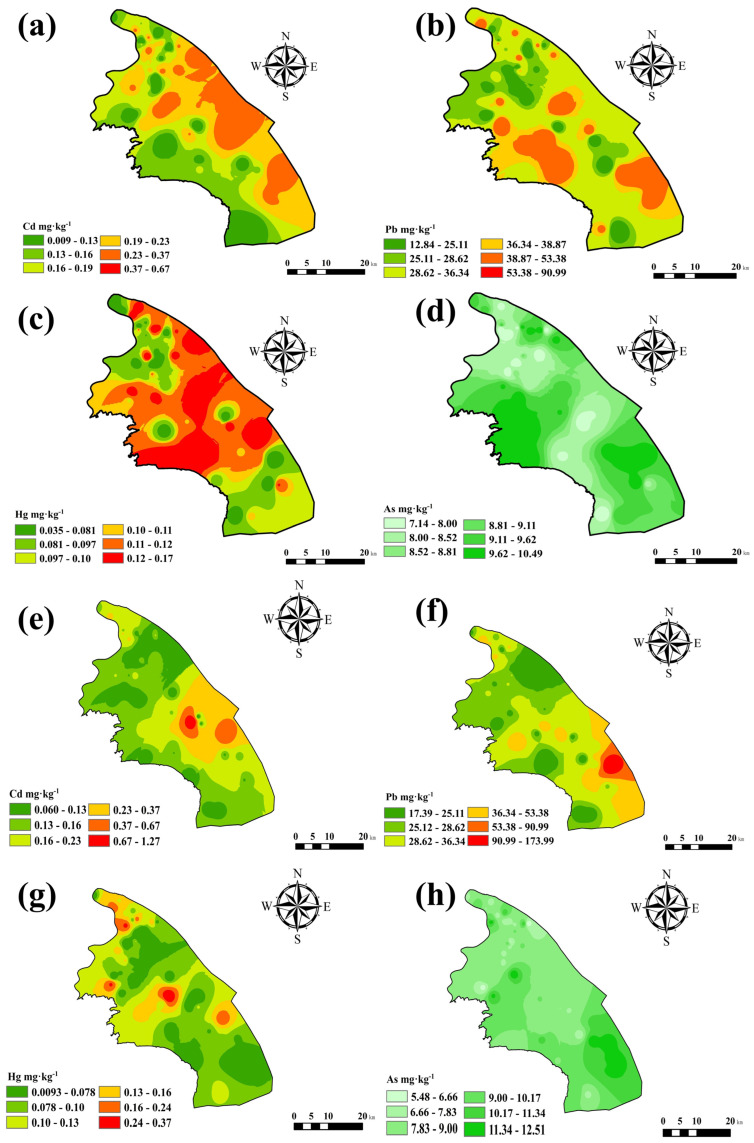
Comparison of the spatial distribution of the measured values of heavy metals in the soil and the predicted values of the RF model. (**a**–**d**) represent the predicted values of Cd, Pb, Hg, and As, respectively. (**e**–**h**) are the measured values of Cd, Pb, Hg and As, respectively.

To further validate the model, spatial interpolation of the RF predictions was applied to the RF-predicted values (Figure 6a–d). The predicted high-concentration areas for Cd were primarily located in the central, eastern, and southern parts of the study area, closely matching the measured Cd spatial distribution. For Pb, the predicted high concentrations appeared in the western and eastern regions, with scattered hotpots in the north. The predicted distribution of Hg revealed a widespread high-concentration zone in the central region. The predicted As distribution was relatively uniform and aligned closely with measured values, with high-value zones in the southern study area. Comparisons between predicted and measured values for these four metals indicate that the RF model achieves high predictive accuracy.

### 3.6. Soil Health Risk Assessment Using Monte Carlo Simulation

**Figure 7:**
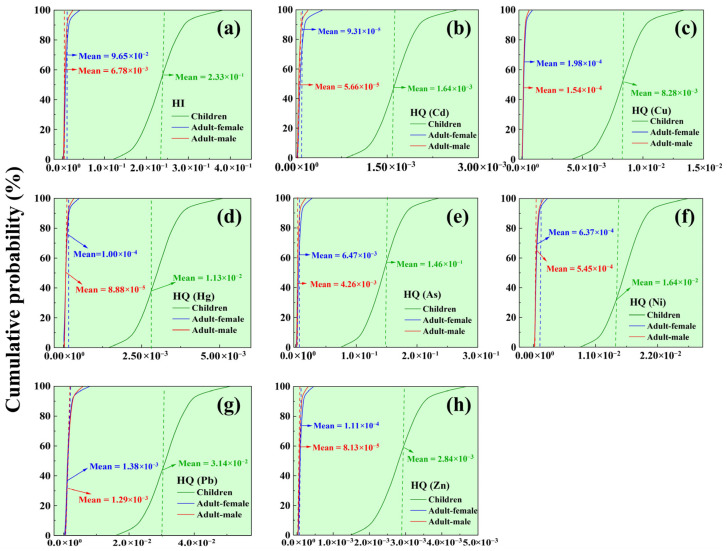
Non-cancer risk for children, adult men and adult women in the study area: (**a**) HI; (**b**) HQ (Cd); (**c**) HQ (Cu); (**d**) HQ (Hg); (**e**) HQ (As); (**f**) HQ (Ni); (**g**) HQ (Pb); and (**h**) HQ (Zn). The dashed vertical lines indicate the mean values for each population group.

A Monte Carlo simulation was employed to derive the probability distributions of the parameters in the HRA model. We assessed the non-carcinogenic and carcinogenic risks of heavy metals in the study area via three main exposure routes (ingestion, skin contact, and particle inhalation) for children, adult males, and adult females (Figure 7 and Table S6). All study populations had negligible non-carcinogenic risks, with HI values for single and multiple heavy metals below 1 (Figure 7a), indicating no potential non-carcinogenic risks in the study area. Children showed relatively higher cumulative non-carcinogenic risks compared to adults. The non-carcinogenic risk patterns for single and multiple heavy metals were similar (Figure 7b–h), with HQ values ordered as children > adult females > adult males, suggesting that heavy metals are unlikely to cause non-carcinogenic risks to human health. These results align with previous studies, such as Wang et al. [77], which reported no potential non-carcinogenic risks from eight heavy metals in farmland soils in Beijing Huairou District.

**Figure 8:**
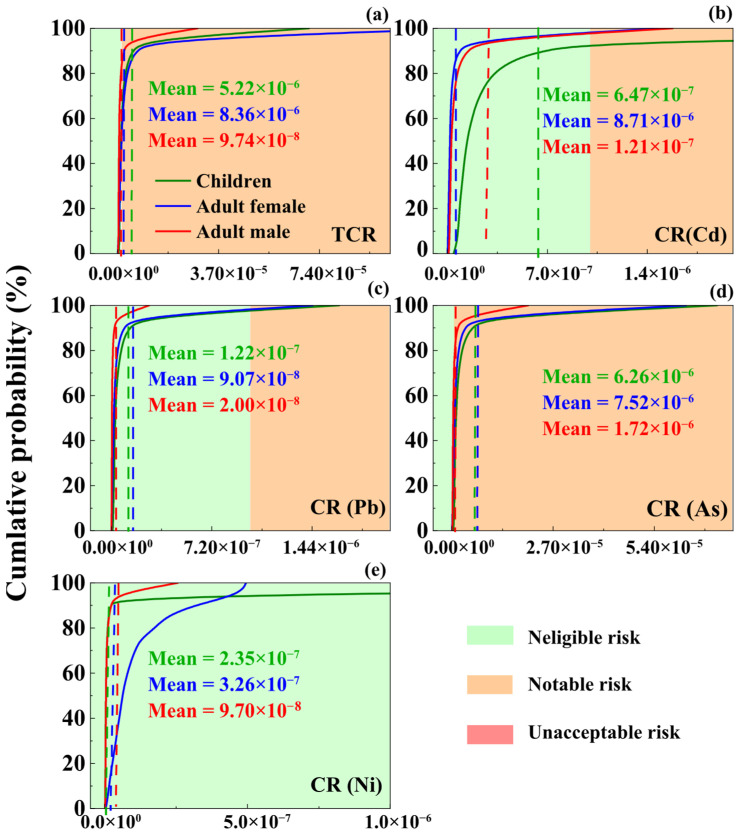
Cancer risk for children, adult men and adult women in the study area: (**a**) TCR; (**b**) CR (Cd); (**c**) CR (Pb); (**d**) CR (As); and (**e**) CR (Ni).

Figure 8 presents the probability distribution of carcinogenic risks. The cumulative probabilities of exceeding the notable risk threshold among the three population groups are 62.1%, 55.7%, and 22.5%, respectively—none surpass the “unacceptable risk” benchmark of 10^−4^. Among the four heavy metals, the carcinogenic risks to humans decrease in the order: As > Cd > Pb > Ni, with Ni posing no carcinogenic risk to any study group. Specifically, the probability of As reaching a notable risk level is 63.5% for children, 56.7% for adult females, and 20.6% for adult males. For Cd, respective probabilities are 5.63% for children, 2.63% for adult females, and 1.86% for adult males. Pb shows a notable risk probability of 2.66% for children and 1.52% for adult females, while posing no significant risk to adult males.

In summary, both non-carcinogenic and carcinogenic risk probability distributions for children, adult females, and adult males in the Pudong New District fall within acceptable thresholds. However, the risk patterns across the three exposure pathways are generally consistent among all population groups, following the order: oral ingestion > inhalation > dermal contact. This indicates that oral ingestion is the dominant exposure route, which aligns with previous studies [77,78]. Additionally, research by Zhao et al. [79] has shown that As poses the highest potential carcinogenic risk to both children and adults. Analysis of the CR calculation formula suggests that this is likely related to As’s high SF. Although PMF results indicate that As in this study primarily originates from natural geological backgrounds, its cumulative probability of carcinogenic risk to children, adult females, and adult males reached 63.51%, 56.74%, and 20.57%, respectively, and As had the highest sensitivity to carcinogenic risk models (contributing 25.02–43.40%). We suggest prioritizing As as an element of concern in subsequent management, with a focus on strengthening the resampling verification and refined risk assessment of As in the 0–0.5 m surface soil.

**Figure 9:**
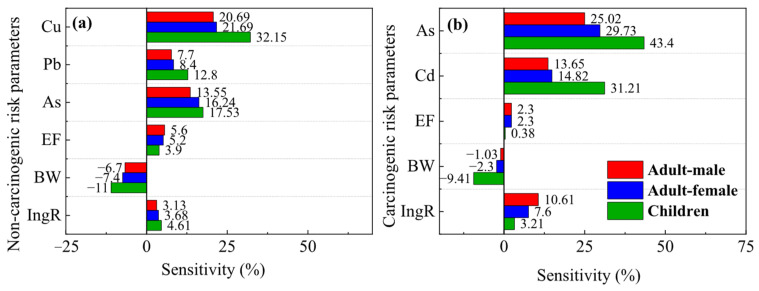
Sensitivity analysis of the HRA model. (**a**) Parameters of the non-carcinogenic risk model; (**b**) parameters of the carcinogenic risk model.

To further assess the impact of model parameters on probabilistic risk assessment results, sensitivity analysis was performed for each parameter. In the non-carcinogenic risk assessment model, children, adult females, and adult males exhibited the highest sensitivity to Cu, accounting for 32.2%, 21.7%, and 20.7%, respectively, followed by As with 17.5%, 16.2%, and 13.6% (Figure 9a). In the carcinogenic risk assessment model, As was the most sensitive heavy metal for children, adult females, and adult males with sensitivities of 43.4%, 29.7%, and 25.0%, respectively, followed by Cd as the second most sensitive at 31.2%, 14.8%, and 13.7% (Figure 9b).

Combining sensitivity analysis with human health risk assessment results (Figure 9), Cu has the highest sensitivity to non-carcinogenic risk models (children: 32.15%; adult females: 21.69%; adult males: 20.69%), followed by As and Pb. The high sensitivity of Cu may be related to its higher concentration in soil (mean 43.36 mg·kg^−1^) and larger coefficient of variation (107.38%), while the oral reference dose of Cu (RfD = 0.04 mg·kg^−1^·d^−1^) is relatively low, making its hazard quotient (HQ) more sensitive to changes in input parameters. As has the highest sensitivity to carcinogenic risk models (43.40% in children, 29.73% in adult women, 25.02% in adult men), followed by Cd, which is closely related to the high carcinogenic slope factor (SF) of As and Cd (oral intake SF = 1.5 mg·kg^−1^·d^−1^, oral intake SF of Cd = 6.1 mg·kg^−1^·d^−1^). Therefore, among the 7 heavy metals, Cu, As, and Cd should be given special attention. Based on the previous source analysis results of this study, although the As element mainly comes from natural geological backgrounds, its human health risks still need to be highly valued. In addition, the IngR (soil ingestion rate) in the model parameters has a certain sensitivity to the model, indicating that reducing oral intake can reduce health risks and protective measures should be taken in daily life. Meanwhile, the sensitivity of BW (body weight) to both carcinogenic and non carcinogenic factors is negative, indicating that appropriate weight gain can help reduce health risks to some extent, which is similar to previous research conclusions [80,81]. In summary, during urban renewal and the redevelopment of related sites in megacities like Shanghai, particular attention should be given to heavy metal exposure, especially for children.

## 4. Limitation of the Study

Although this study systematically revealed the pollution characteristics, sources, and health risks of heavy metals in soils from typical redevelopment areas in the Pudong New Area, several limitations still exist. First, regarding the sampling layout, although 52 plots were covered and 516 samples were collected, the sample is not fully representative of all types of redevelopment land in the Pudong New Area due to constraints related to land use types and industrial distribution. Therefore, caution is needed when extrapolating the results. Next is the sampling depth. In this study, fixed depth stratified sampling (0–0.5 m, 0.5–1.5 m, ≥1.5 m) was used instead of sampling based on the soil occurrence layer. Although fixed depth sampling is currently the standard method for pollution investigation of construction land in China, and the soil in the study area is mostly industrial disturbed soil, the natural profile structure has been destroyed. However, this method may overlook the enrichment effect of organic matter layers on heavy metals. Second, the study only measured seven elements (Cd, Pb, Cu, Zn, Ni, Hg, and As) and did not include other potentially toxic heavy metals such as chromium (Cr) and cobalt (Co), nor typical organic pollutants, which may lead to an underestimation of the ecological and health risks associated with combined pollution. Third, this investigation was based on a single cross-sectional sampling campaign and lacks time-series data, making it impossible to reveal the temporal and spatial dynamics of heavy metal concentrations or their seasonal migration and transformation patterns. Finally, regarding the health risk assessment, although Monte Carlo simulation was introduced to reduce parameter uncertainty, the exposure parameters used were primarily derived from the literature rather than from local measured data, which may cause deviations between the risk assessment results and the actual situation. Moreover, the current model has not fully considered potential interactions among multiple heavy metals (e.g., synergistic or antagonistic effects) or the combined effects of multi-pathway exposure. Future research should integrate long-term monitoring and multi-media sampling, expand the range of pollutants, enhance speciation analysis and bioavailability assessment of heavy metals, introduce localized exposure parameters, and further improve the accuracy and scientific validity of risk assessment through multi-model coupling. This will provide more targeted scientific support for soil environmental management in the context of urban renewal.

## 5. Conclusions

This study investigated heavy metal contamination in soils from typical industrial sites in Pudong New District, Shanghai, focusing on concentration characteristics, spatial patterns, risk levels, and pollution sources. The main conclusions are as follows:

(1) Except for As, the mean concentrations of Cd, Pb, Cu, Zn, Ni, and Hg in surface soils exceeded background values by 1.04–1.81 times, and concentrations generally decreased with depth. Spatially, Pb and Hg were concentrated in the central area; Cd and Zn were widely distributed; Cu and Ni showed similar central patterns; and As was relatively uniform.
(2) The geo-accumulation index followed the order: Zn > Cu > Ni > Cd > Pb > As > Hg. Overall contamination was mild, with localized moderate to heavy pollution. The mean comprehensive potential ecological risk index was 112.78, indicating medium ecological risk.
(3) The random forest model demonstrated good predictive performance for Cd, Pb, Hg, and As (R^2^ > 0.8), and predicted spatial patterns were consistent with measured distributions.
(4) Correlation analysis combined with PMF identified transportation, chemical combustion, metal industry, and natural geological sources as the main contributors. Transportation dominated Cd and Zn; chemical combustion dominated Pb and Hg; metal industry dominated Cu and Ni; and natural sources dominated As.
(5) Monte Carlo–based health risk assessment indicated no significant non-carcinogenic risk for any population group. However, cumulative probabilities of reaching notable risk levels were 62.1% (children), 55.7% (adult females), and 22.5% (adult males). Ingestion was the primary exposure pathway. Although As mainly derives from a natural background, its sensitivity to cancer risk models is the highest (25.02–43.40%), and the cumulative probability of cancer risk in children exceeding 1 × 10^−4^ is 63.51%. In subsequent management, priority should be given to strengthening the fine management of arsenic.

## Figures and Tables

**Table 1:** Reference of the evaluation standard of soil heavy metals.

	Cd	Pb	Cu	Zn	Ni	Hg	As
Soil background values of Shanghai (mg·kg^−1^)	0.13	25.5	28.6	86.1	31.9	0.10	9.10
Toxicity coefficient	30	5	5	1	5	40	10

**Table 2:** Soil heavy metal contents of different depths in the study area.

Element	Sampling Depth	Max (mg·kg^−1^)	Min (mg·kg^−1^)	Mean ± Standard Deviation (mg·kg^−1^)	CV (%)	BV
Cd	Surface layer	1.27	0.06	0.17 ± 0.16	100	0.13
	subsurface layer	0.61	0.05	0.15 ± 0.11	75.1	
	Saturated zone	0.53	0.05	0.11 ± 0.09	79.0	
Pb	Surface layer	174	17.3	30.8 ± 21.4	69.4	25.5
	subsurface layer	169	15.6	27.7 ± 27.7	74.7	
	Saturated zone	30.8	14.2	21.5 ± 4.15	19.3	
Cu	Surface layer	336	15.0	43.4 ± 43.2	107.4	28.6
	subsurface layer	79.9	15.1	33.7 ± 13.6	40.7	
	Saturated zone	51.3	12.8	26.4 ± 8.20	31.0	
Zn	Surface layer	827	29.8	156 ± 127	81.2	86.1
	subsurface layer	810	29.0	135 ± 153	113	
	Saturated zone	380	28.3	91.4 ± 62.4	68.3	
Ni	Surface layer	130	11.6	44.7 ± 53.1	119	31.9
	subsurface layer	88.5	24.3	35.7 ± 43.1	149	
	Saturated zone	62.9	17.90	32.6 ± 33.1	102	
Hg	Surface layer	0.37	0.03	0.11 ± 0.07	66.28	0.10
	subsurface layer	0.20	0.01	0.08 ± 0.04	52.09	
	Saturated zone	0.14	0.01	0.06 ± 0.02	16.67	
As	Surface layer	12.6	5.39	8.87 ± 1.74	19.66	9.10
	subsurface layer	11.1	5.17	8.76 ± 1.82	20.76	
	Saturated zone	10.1	4.73	7.74 ± 1.64	21.21	

**Table 3:** Potential Ecological Risk Assessment Results (*E_i_* and *RI*) for Surface Soils.

	Evaluation Index of Potential Ecological Risks (*E_i_*)							*RI*
	Cd	Pb	Cu	Zn	Ni	Hg	As	
Max	293	34.2	58.8	9.60	20.4	146.8	13.8	402
Min	13.6	3.39	2.63	0.35	1.82	3.50	5.92	48.2
AVA	39.9	6.04	7.58	1.81	7.00	40.7	9.75	113

## Data Availability

The original contributions presented in this study are included in the article/Supplementary Materials. Further inquiries can be directed to the corresponding authors.

## Supplementary Materials

The following supporting information can be downloaded at: https://www.mdpi.com/article/10.3390/toxics14040315/s1. Text S1. Formulas for calculating the average daily intake dose (ADD) in health risk assessment. Table S1. Laboratory method detection limit for heavy metal elements. Table S2. Classification standard of geo-accumulation index (*I_geo_*). Table S3. Classification criteria of soil heavy metal potential ecological risk. Table S4. Probability distribution functions of the parameters in health risk assessment model. Table S5. Reference dose (RfD) and lope factor (SF) values of the different exposure pathways of soil HMs. Table S6. Summary satistics for non-carcinogenic and non-carcinogenic health risk range.
