# Supplementary material for: Spatial Distribution, Source Apportionment and Risk Assessment of Heavy Metal Pollution in Typical Redevelopment Sites in Pudong New District, Shanghai
Source: Toxics. 2026 Apr 8;14(4):315. doi: 10.3390/toxics14040315 (PMC13119855; doi:10.3390/toxics14040315)
Supplement: Supplementary file 1 [file toxics-14-00315-s001.zip › toxics-4233811-supplementary.pdf]

Supplementary Information for

Spatial distribution, source apportionment and risk assessment of heavy metal pollution in typical redevelopment sites in Pudong New District, Shanghai

Cheng Shen[a,b], Jian Wu[c,*], Ye li[d,*]

[a] State Environmental Protection Engineering Center for Urban Soil Contamination Control and Remediation, Shanghai Academy of Environmental Sciences, Shanghai 200233, China

[b] State Environmental Protection Key Laboratory of Environmental Risk Assessment and Control on Chemical Process, School of Resources and Environmental Engineering, East China University of Science and Technology, Shanghai 200237, China

[c] Shanghai Technology Center for Reduction of Pollution and Carbon Emissions, Shanghai 200235, China

[d] Key Laboratory of Geographic Information Science (Ministry of Education), School of Geographic Sciences, East China Normal University, 500 Dongchuan Road, Minhang District, Shanghai 200241, China

Corresponding Authors:

E-mail address: wuj@saes.sh.cn (Jian Wu); Yli@geo.ecnu.edu.cn (Min Liu).

This file includes:

Number of pages: 9
Number of Tables:6
Number of Text: 1

Contents

Texts..3

Text S1. Formulas for calculating the average daily intake dose (ADD) in health risk assessment**...3**

Tables 4

Table S1. Laboratory method detection limit for heavy metal elements. 4

Table S2. Classification standard of geo-accumulation index (I_{geo}). 5

Table S3. Classification criteria of soil heavy metal potential ecological risk. 5

Table S4. Probability distribution functions of the parameters in health risk assessment model. 6

Table S5. Reference dose (RfD) and lope factor (SF) values of the different exposure pathways of soil HMs. 7

Table S6. Summary satistics for non-carcinogenic and non-carcinogenic health risk range....8

References..9

Texts

Text S1. Formulas for calculating the average daily intake dose (ADD) in health risk assessment

Loading the "Crystal Ball" add-in in Excel and editing the formulas for non-carcinogenic risk and carcinogenic risk, proceed with the "two-dimensional simulation".The average daily intake dose (ADD, $mg \cdot kg^{-1} \cdot day^{-1}$) of a contaminant through direct ingestion, dermal contact and inhalation absorption was calculated as follows:

$$ADD_{ing} = \frac{C \times IngR \times EF \times ED}{BW \times AT} \times 10^{-6} \quad (1)$$

$$ADD_{inh} = \frac{C \times InhR \times EF \times ED}{PEF \times BW \times AT} \quad (2)$$

$$ADD_{der} = \frac{C \times SA \times AF \times ABF \times EF \times ED}{BW \times AT} \times 10^{-6} \quad (3)$$

where C is the concentration (mg/kg) of heavy metal concentration in soil; the detailed explanations of these variables and their real values for people are defined in Table S4.

Tables

Table S1. Laboratory method detection limit for heavy metal elements.

Elements	Measurement method	Detection limit mg·kg^{-1}	Main instruments
Cd	Soil and sediment-Determination of aqua regia extracts of 12 metal elements-Inductively coupled plasma mass spectrometry （HJ 803-2016）	0.03	ICP-MS Agilent 7900
Pb		0.10	
Cu		1.00	
Zn		0.50	AAS ZA3000
Ni		5.00	ICP-MS Agilent 7900
As	Soil and sediment — Determination of mercury, arsenic, selenium, bismuth, antimony — Microwave dissolution/Atomic Fluorescence Spectrometry （HJ 680-2013）	0.01	AAS ZA3000
Hg		0.002	AFS-9330

Table S2. Classification standard of geo-accumulation index (I_{geo}).

Level	I_{geo}	Pollution Level
1	$I_{geo} \leq 0$	No Pollution
2	$0 < I_{geo} \leq 1$	low pollution
3	$1 < I_{geo} \leq 2$	Moderate Pollution
4	$2 < I_{geo} \leq 3$	Moderately Heavy Pollution
5	$3 < I_{geo} \leq 4$	Heavy Pollution
6	$I_{geo} \geq 4$	Extremely Heavy Pollution

Table S3. Classification criteria of soil heavy metal potential ecological risk.

Level	E_i	Risk Level	RI	Risk Level
1	$E_i \leq 40$	Minor Risk	$RI \leq 110$	Minor Risk
2	$40 < E_i \leq 80$	Moderate Risk	$110 < RI \leq 220$	Moderate Risk
3	$80 < E_i \leq 160$	Considerable Risk	$220 < RI \leq 440$	Considerable Risk
4	$160 < E_i \leq 320$	High Risk	$440 < RI \leq 660$	High Risk
5	$E_i > 320$	Extreme Risk	$RI > 660$	Extreme Risk

Table S4. Probability distribution functions of the parameters in health risk assessment model.

Parameter	Description	Unit	Distribution Type	Children	Adult Female	Adult Male	Reference(s)
IngR	Soil Ingestion Rate	mg/day	Triangular distribution	200	100	100	[1]
InhR	Inhalation Rate	m3/day	Normal distribution	8.6	14	19	[2]
EF	Exposure Frequency	days/year	Triangular distribution	350			[2]
ED	Exposure Duration	years	Uniform distribution	6	24		[2]
BW	Body Weight	kg	Normal distribution	29.3	59.59	67.55	[1, 2]
AT	Average Time for Carcinogenic Risk	days	Point distribution	365×70			[2]
	Average Time for Carcinogenic Risk	days	Uniform	365×ED			[1, 2]
SA	Exposed Skin Surface Area	cm2	Point	2300	4900	5400	[3]
AF	Soil Adherence Factor	mg/cm2	Point	0.2			[3]
PEF	Particulate Emission Factor	m3/kg	Point	1.36E+09			[1-3]

Table S5. Reference dose (RfD) and lope factor (SF) values of the different exposure pathways of soil HMs.

	RfD ($mg \cdot kg^{-1} \cdot d^{-1}$)			SF ($(mg \cdot kg^{-1} \cdot d^{-1})^{-1}$)			Reference
	Ingestion	Dermal contact	Inhalation	Ingestion	Dermal contact	Inhalation	
Cu	0.04	0.012	0.0402	-	-	-	[2]
Cr	0.003	0.00006	0.0000286	0.0085	-	42	[1, 2]
Ni	0.02	0.0054	0.00009	-	-	0.84	[3]
Zn	0.3	0.06	0.3	-	-	-	[3]
Pb	0.0035	0.000525	0.00352	0.0085	-	-	[3]
Cd	0.001	0.00001	0.00001	6.1		6.3	[1-3]
As	0.0003	0.0003	0.000123	1.5	3.66	15.1	[3]
Hg	0.0003	0.000021	0.0000857	-	-	-	[1, 2]

Table S6. Summary satistics for non-carcinogenic and non-carcinogenic health risk range.

Risk	HMs	Mean											
		Children	Adult-female	Adult-male	Children			Adult-female			Adult-male		
					ingestion	dermal	inhalation	ingestion	dermal	inhalation	ingestion	dermal	inhalation
	Cd	2.13E-03	1.27E-04	2.75E-05	1.93E-03	4.56E-08	1.98E-04	1.23E-04	9.85E-08	4.22E-06	2.62E-05	2.55E-08	1.22E-06
	Hg	3.62E-03	2.30E-04	4.91E-05	1.08E-03	2.55E-08	1.11E-04	6.89E-05	5.52E-08	2.37E-06	1.47E-05	1.43E-08	6.84E-07
	Pb	5.38E-02	1.35E-03	1.29E-03	2.37E-01	5.60E-06	2.43E-02	1.51E-02	1.21E-05	5.19E-04	3.22E-03	3.13E-06	1.50E-04
HQ	Cu	2.46E-03	2.60E-04	2.38E-04	3.24E-01	7.64E-06	3.31E-02	2.06E-02	1.65E-05	7.08E-04	4.40E-03	4.27E-06	2.05E-04
	Zn	3.32E-03	2.11E-04	4.50E-05	9.95E-01	2.34E-05	1.02E-01	6.33E-02	5.07E-05	2.17E-03	1.35E-02	1.31E-05	6.29E-04
	As	1.42E-01	1.31E-02	2.79E-03	6.15E-02	1.45E-06	6.29E-03	3.91E-03	3.13E-06	1.34E-04	8.34E-04	8.11E-07	3.89E-05
	Ni	1.46E-02	1.08E-03	2.36E-04	3.15E-01	7.42E-06	3.22E-02	2.00E-02	1.6E-05	6.87E-04	4.27E-03	4.15E-06	1.99E-04
HI	Total	2.22E-01	1.64E-02	4.68E-03					/				
	Cd	8.00E-07	8.71E-08	2.20E-07	8.00E-07	3.81E-11	5.07E-11	8.60E-08	2.17E-12	1.04E-09	8.78E-09	2.80E-12	4.22E-12
CR	Pb	1.37E-07	1.48E-08	3.76E-08	9.83E-05	4.68E-09	6.23E-09	1.06E-05	2.67E-10	1.28E-07	2.19E-07	6.99E-11	1.06E-10
	As	6.26E-06	7.52E-07	1.72E-06	2.55E-05	1.21E-09	1.61E-09	2.74E-06	6.90E-11	3.32E-08	2.70E-05	8.59E-09	1.30E-08
	Ni	1.10E-09	2.27E-08	2.29E-09	1.30E-04	6.20E-09	8.26E-09	1.40E-05	3.53E-10	1.70E-07	6.98E-06	2.22E-09	3.36E-09
TCR	Totall	7.20E-06	8.77E-07	1.98E-06					/				

References

[1] Z. Sun, Y. Hu, H. Cheng, Public health risk of toxic metal(loid) pollution to the population living near an abandoned small-scale polymetallic mine, Science of The Total Environment, 718 (2020) 137434.

[2] H. Luo, Q. Wang, Q. Guan, Y. Ma, F. Ni, E. Yang, J. Zhang, Heavy metal pollution levels, source apportionment and risk assessment in dust storms in key cities in Northwest China, Journal of Hazardous Materials, 422 (2022) 126878.

[3] L. Wu, W. Yue, J. Wu, C. Cao, H. Liu, Y. Teng, Metal-mining-induced sediment pollution presents a potential ecological risk and threat to human health across China: A meta-analysis, Journal of Environmental Management, 329 (2023) 117058.
